# Etiology of bacterial meningitis: a cross-sectional study among patients admitted in a semi-urban hospital in Nairobi, Kenya

**DOI:** 10.11604/pamj.supp.2017.28.1.9383

**Published:** 2017-11-04

**Authors:** Charles Njonjo Gituro, Andrew Nyerere, Musa Otieno Ngayo, Edward Maina, Jane Githuku, Waqo Boru

**Affiliations:** 1 National Public Health Laboratory Services (NPHLS), Ministry of Health, Nairobi Kenya,; 2 Institute of Tropical Medicine and Infectious Diseases, Jomo Kenyatta University of Agriculture and Technology, Nairobi, Kenya; 3 Field Epidemiology and Laboratory Training Program, Nairobi, Ministry of Health, Kenya,; 4 Centre of Microbiology and Research Kenya Medical Research Institute, Nairobi, Kenya

**Keywords:** Bacterial meningitis, etiology, associated factors, Kenya

## Abstract

**Introduction:**

bacterial meningitis, responsible for childhood morbidity and mortality, can also lead to permanent neurological disability among survivors. This study conducted from January to December, 2015 used standard bacteriological and molecular methods to investigate the etiology of three common causes of bacterial meningitis among hospitalized patients admitted at a semi-urban hospital in Nairobi, Kenya.

**Methods:**

a total of 196 patients admitted at Mama Lucy Kibaki with clinically diagnosed meningitis were recruited into this cross-sectional study. Participants’ information was collected through patient interviews and abstraction of health records. Bacterial culture, gram stains and multiplex polymerase chain reaction (PCR) were used to investigate causes of bacterial meningitis from cerebrospinal fluid (CSF) samples. Characteristics such as age, gender, occupation, underlying conditions of patients with laboratory confirmed bacterial meningitis infection are described.

**Results:**

among the 196 patients diagnosed with bacterial meningitis, the median age was 1 year (range 1 to 36 years) with 87.2% aged 1 to 4 years; 54.6% were males. Using PCR, 22 out of 196 (11.2%) samples had evidence suggesting a bacterial infection. These included 12/22 (54.5%) *S. pneumonia*, 7/22 (31.8%) *N. meningitides* and 3/22 (13.6%) *H. influenza*. From bacterial culture, four of 196 (2.1%) samples grew *S. pneumonia*. All three samples found positive for *H. influenza* were from male patients aged between 1 to 4 years.

**Conclusion:**

of the three common causes evaluated, *S. pneumonia* was the most common cause of bacterial meningitis among patients from this region, particularly among infants. One older patient was diabetic, thereby highlighting the importance of pre-existing conditions. Although serotyping of bacteria was not done, under-vaccination might have played a role in the cases identified and ensuring complete and timely vaccination may prevent further cases of bacterial meningitis.

## Introduction

Meningitis is an infection of the membranes and cerebrospinal fluid (CSF) surrounding the brain and spinal cord. It is a severe disease of serious public health importance that is responsible for substantial morbidity and mortality and may be caused by infection with a large variety of bacteria, viruses, fungi, or parasites [1]. Bacterial meningitis requires prompt recognition and treatment [2]. Accurate and timely identification of the etiological agents that cause bacterial meningitis and the populations at risk of bacterial meningitis is important to initiate public health measures and ensure appropriate management [3].

The epidemiology of bacterial meningitis varies by country and depends on factors such as the availability and use of vaccines against various etiologies and other risk factors associated with immune status of individuals who become infected. In the Middle East, *N. meningitides* (30%), *H. influenza* (20%) and *S. pneumonia* (50%) have been reported as the predominant pathogens [4, 5]. The most common agents in adults and children are *S. pneumonia* and *N. meningitides*, because vaccination has virtually eliminated *H. influenza* type b (Hib) meningitis in children where Hib vaccine is given [6, 7]. Studies among children and adults have identified factors associated with bacterial meningitis infection including being younger than one year of age due to a naive/immature immune system, waning of protective maternal antibody levels and exposure to young adult carriers in the household [8]. Close and prolonged contact with a carrier, household crowding, travelling to countries with epidemic area are also risk factors for infection. Alcoholism, human immunodeficiency virus (HIV) infection, diabetes mellitus, the use of immunosuppressive drugs and cancer may cause dysfunction of the immune system and thereby increase the risk of invasive infections, including meningitis [9].

In developing countries, *S. pneumonia* has been identified as a leading cause of meningitis among children and adults [10-12]. In Kenya, a study conducted in 2015 among HIV-infected Kenyan patients in the year before pneumococcal conjugate vaccine (PCV) introduction showed 43% of them were infected with Streptococcus pneumonia [11]. Further studies evaluating etiology and correlates of bacterial meningitis in Kenya are vital for case management. Accurate laboratory identification of the etiology of bacterial meningitis is essential to provide optimal patient therapy, appropriate case management, effective public health actions and information upon which to base decisions regarding immunization programs [2, 3]. We investigated three important etiologies of bacterial meningitis (*S. pneumonia*, N. Meningitidis, and *H. influenza*e) among patients with meningitis-like symptoms admitted at Mama Lucy Kibaki Hospital, a county hospital serving a large population of low-income earners, inhabitants of Nairobi, Kenya.

## Methods

### Study design, setting and case definition

This pilot surveillance program for bacterial meningitis was carried out at Mama Lucy Hospital, in Nairobi the most populated, capital city of Kenya. The hospital serves mainly low-income community members. On a monthly basis, the hospital provides care for ~ 28,000 outpatients, ~ 1,200 inpatients and ~ 750 deliveries at a minimum every month. Patients of all ages presenting with meningitis-like symptoms including fever, severe and persistent headache, neck stiffness, seizures were identified as suspected cases of meningitis.These patients were requested to provide informed consent or assent if minors and asked if they were available for a 20-minute interview.

### Sampling strategy

**Figure 1:**
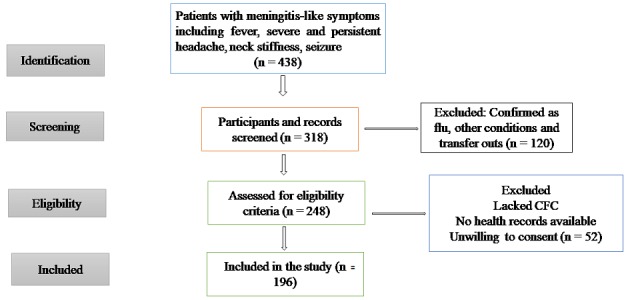
flow chart of participant’s recruitment, data collection and spatial analysis

Because there were not sufficient resources for laboratory testing of all suspected cases of bacterial meningitis, a systemic sampling method was used to select patients for enrollment. We created a sampling frame using the laboratory register, which contained 438 entries of patients that met the case definition, as obtained from the nursing desk which included the period of January to December 2015 (Figure 1). Between 20 and 25 samples were collected per month between the months of May to December 2015.

### Data collection

### Interviews and health record review

Patients enrolled by the resident doctor had their medical files reviewed to obtain information on: HIV status, medical history, vaccination history and meningitis signs and symptoms. Symptoms of meningitis included: fever, severe persistent headache, neck stiffness, nausea and vomiting, confusion and disorientation, drowsiness or sluggishness, sensitivity to bright light, poor appetite and more severe symptoms including seizure and coma. When able to respond, adult patients or guardians of minor patients were interviewed using structured questionnaires to gather information such as: demographic characteristics, healthcare access, onset of ailment, contact and possible mode of infection, social support and confirmation of vaccination history when the reports were missing from the charts.

### Laboratory test

The lumbar puncture technique was used to collect cerebrospinal fluid (CSF) into three tubes for chemistry, microbiology and cytology which is a standard procedure done for all patients with suspected meningitis at the hospital. CSF specimens were transported to the National Microbiology Reference Laboratory located at National Public Health Laboratory Services complex within thirty minutes of collection. Two ml of CSF were dispensed into cryotubes and stored at -80°C until further testing. The methods described by Berkley et al., 2001 [13] were used to analyze the CSF. The sample was centrifuged for 10-15 minutes at 1000 rpm, 1-2 drops of the well-mixed CSF sediment were placed on the slide and air dried. The smears were fixed using 95% methanol, stained using the standard method and examined with 100X oil immersion objective. CSF specimens were cultured using standard techniques described by Berkley et al., 2005 [14]. About 20μl of CSF were inoculated onto plates of 7% sheep-blood agar and 5% chocolate blood agar and incubated at 36.5°C for 18-24h in a candle jar.

Plates without visible signs of growth were incubated for additional 24 hours. The bacterial identification was done using the guidelines described by Garcia et al. [15]. The bacterial DNA were extracted from the last portion of CSF samples using QIAGEN DNA extraction kit (Hilden, Germany) according to the kit manufacturer’s instructions. Amplification was done using semi-nested multiplex PCR using universal primers for the bacterial 16s rDNA, and specific primers for *S. pneumonia* (STREP), *H. influenza* (HI) and *N. meningitides* (NM), in a 25μl reaction volume the amplification condition (using AB 9700 Thermocylcer, USA) as follows: 5 minutes initial denaturation at 94°C, 35 cycles of denaturation for 30 seconds at 94°C, annealing for 30 seconds at 55°C and extension for 30 seconds at 72°C, and a final extension incubation for 15 minutes at 72°C. Detection was done using ethidium bromide stained 1.5% agarose gel electrophoresis under ultraviolet rays at 300 nm in an HP AlphaImager® (Alpha Immutech/South Africa). The PCR primers did not differentiate the serotypes of the bacteria.

### Data management and analysis

Prior to any protocol-related procedures being conducted, patients were assigned unique identification numbers (UIN) to allow linkage between information from questionnaires and biological specimens. All patient’s information was entered into the study databases (Microsoft excel and STATA files) associated with a UIN in password protected files. Double entry systems for the data were maintained. The questionnaires records were kept in a locked filing cabinet located in a restricted-access room at the research station. Frequency and percentages were calculated for characteristics such as age, gender, occupation, underlying conditions of patients with laboratory confirmed bacterial meningitis infection. All statistical analyses were performed using STATA version 13 (StataCorp LP, Texas, USA). Crowding was defined as more than five people in a one to two roomed house while spatially crowded was defined as less than 3 people in one to two roomed house. An infant was defined as ages 1 to 28 days, a child was defined as one month to 18 years of age and an adult was a patient age greater than 18 years.

### Ethical consent and approvals

Consent was obtained from the patients or guardian for the minors by the research nurse. Approval to conduct the study was granted by the hospital medical super intendent before commencement of recruitment exercise. This study was reviewed and approved by the Jaramogi Oginga Odinga Teaching and Referral Hospital-Ethics & Research Committee (ERC.IB/VOL.1/167).

## Results

During recruitment of participants, 318 were selected, 248 were eligible and 196 were enrolled for the study (Figure 1). All the 196 participants or their guardians, where applicable, consented to participate in the study and CSF was collected (Table 1). Patients were 55% male. The median age of the participants was one year (range, 1 to 37 years). About 53% of participants resided in urban informal settlements. The median household size was 4 persons (range, 4 to 7 persons). There were 99% participants with fever, 70.4% with stiff neck, 75% were irritable, 68.9% were drowsy, 90.8% were vomiting; 3.1% of the participants were identified as HIV positive. Using PCR, 22 out of 196 (11%) samples had evidence of a bacterial infection. These included 12 of 22 (55%) *S. pneumonia*, 7 of 22 (32%) *N. meningitides* and 3 of 22 (14%) *H. influenza*. The three pathogens shared similar seasonality being identified between the months of June to November. Of the seven samples positive for *N. meningitides*, five were from female patients. All seven of the patients with *N. meningitides* by PCR were aged between 1 to 4 years (Table 1). None of the patients had received meningococcal vaccine. Four of those with positive *S. pneumonia* using culture were also positive by PCR. Seven patients with evidence of *S. pneumonia* had received at least one dose of Pneumococcal Conjugated Vaccine (PCV10) while 5 were completely unvaccinated.

**Table 1:** distribution in the etiology of bacterial meningitis across patients’ characteristics

Variable	Sample size (N = 196)	Type of Bacteria identified			
		Negative (N = 174)	*Haemophilus influenza* (N = 3)	*Neisseria meningitides* (N = 7)	*Streptococcus pneumonia* (N = 12)
**Gender**					
Male	107 (54.6)	97 (49.5)	3 (1.5)	2 (1.1)	5 (2.6)
Female	89 (45.4)	77 (32.3)	0	5 (2.6)	7 (3.6)
**Age**					
Infant	144 (73.5)	126 (64.3)	2 (1.1)	5 (2.6)	11 (5.6)
Children	23 (11.7)	19 (9.7)	1 (0.5)	2 (1.1)	1 (0.5)
Adult	7 (3.6)	7 (3.6)	0	0	0
**Region**					
Crowded	104 (53.1)	89 (45.4)	2 (1.1)	4 (2.1)	9 (4.6)
Spatially crowded	92(46.9)	85(43.4)	1(0.5)	3 (1.5)	3 (1.5)
**Household size**					
≤4	129 (65.8)	117 (59.7)	1 (0.5)	3 (1.5)	8 (4.1)
≥5	67 (34.2)	57 (29.1)	2 (1.1)	4 (2.1)	4 (2.1)
**Household rooms**					
1-2	139 (70.9)	125 (63.8)	2 (1.1)	5 (2.6)	7 (3.6)
>3	57 (29.1)	49 (25)	1 (0.5)	2 (1.1)	5 (2.6)
**Pneumonia**					
Yes	51 (26.1)	42 (21.4)	2 (1.1)	4 (2.1)	3 (1.5)
No	145 (73.9)	132 (67.3)	1 (0.5)	3 (1.5)	9 (4.6)

From bacterial culture, four of 196 (2.1%) CSF specimens grew *S. pneumonia*. Out of 12 samples found positive for *S. pneumonia*, eleven were from patients aged between 1 to 4 years and seven were detected from female patients. One participant with *S. pneumonia* infection had diabetes (Table 1). Household size and household crowding characteristics are given in Table 1. All three specimens positive for *H. influenza* were from male patients, among whom 2 were 1 year and 1 was 4 years old and one of these patients with *H. influenza* were HIV infected. Two out of three *H. influenza* positive specimens came from patients who had pneumonia (Table 1). The two 1-year-old patients with *H. influenza* had not been vaccinated against Haemophilus influenza type b (Hib).

## Discussion

The findings are consistent with other findings: that young and unvaccinated children (aged 1 to 4 years) often present with bacterial meningitis because of their immunologically vulnerable state [16]. This study detected the three important bacteria etiologies in 11% cases of suspected bacterial meningitis cases. Various prevalence rates of bacterial meningitis have been reported elsewhere in Kenya, which may relate to different study designs, as well as changes in epidemiology and prevention efforts, including vaccination programs. In Kilifi, Kenya, a 2011 study reported a 6% prevalence of bacterial meningitis in all hospital admissions [17] with the most common bacterial etiologies *S. pneumonia*, Group B Streptococcus and *H. influenza* [17]. In Western Kenya a study found *S. pneumonia* at a prevalence of 38.6 % was the leading cause of meningitis among infants aged < 60 days in 2011 [17]. In 2013 the largest tertiary hospital in Nairobi, Kenya [18] reported 20% of confirmed cases of bacterial meningitis were caused by *S. pneumonia*.

This study did not identify other possible etiologies of our meningitis cases that include other bacterial organisms such as Sphingobacterium multivorum [19], Tuberculous meningitis [20], Aeromonas hydrophila [21], or fungal species (Candida albicans, Cryptococcus sps) [22], or viral etiologies including enteroviruses, mumps virus, herpes viruses, influenza viruses, cytomegalovirus (CMV) and Epstein-Barr Virus (EBV). Enteroviruses are responsible for about 90% of aseptic meningitis worldwide, especially in underdeveloped countries with poor sanitation and low standards of hygiene [23, 24].

Our study reported *S. pneumonia* as the most common etiology, followed by *N. meningitides* and *H. influenza* as a causative agent of bacterial meningitis, the study did not include the serotypes of the pathogens. Similar reports of bacterial infection in neonates and young infants in 2010 and 2011 showed the predominance of *S. pneumonia* as the most common etiology of bacterial meningitis have been documented in other regions of Kenya [17, 25]. In the “meningitis belt” of Sub-Saharan Africa, comprising of 26 countries from Senegal in the west to Ethiopia in the east before 2010, *N. meningitides* was responsible for the large majority of bacterial meningitis epidemics [26]. The 2010 progressive introduction of a meningococcal A conjugate vaccine in the 26 countries in the African meningitis belt has brought about a dramatic reduction of *N. meningitides* with the relative proportion of cases due to *S. pneumonia* rising [27]. The meningococcal conjugate vaccine against group A was introduced in Africa in 2010 with some parts of Kenyan population (Coastal Kenya) who were vaccinated. However, none of the Study participant had received meningococcal vaccine. Pneumococcal conjugate vaccine (PCV) given as PVC10 was introduce in the Kenya vaccination program in 2011 and coverage is estimated at 54% by the Ministry of Health. Vaccine against H influenza type b was introduced in 2001 delivered in combination with diphtheria, tetanus, pertussis and hepatitis B as pentavalent (DTP-Hib-Hep) and is given in three doses to the children at the age of six, ten and fourteen weeks. DTP-Hib-Hep Coverage was estimated at 52% as at July 2017 by the Kenya expanded Programme of immunization but there was high dropout rate for children who did not finish all the three doses.

Differences in the epidemiology of bacterial meningitis relates to factors including geographical conditions and presence of vaccination programs for Meningococcal, *H. influenza* type B and *S. pneumonia* [28]. The current study occurred in only one site and therefore results are not necessarily generalizable to the whole country. The cases were isolated between the months of June to November, which is a relatively wet season in Kenya. Bacterial meningitis is more common during dry seasons, in rural areas and in crowded informal urban settlements [29]. One patient was diabetic with bacterial meningitis infection in this study Bacterial infection have been shown to contribute about 49% of the diabetic ketoacidosis cases [30, 31]. Diabetic ketoacidosis is a life-threatening acute complication of type 1 diabetes mellitus. The associations of diabetic ketoacidosis with tuberculous meningitis and group B streptococcal meningitis have been reported [30, 31].

Several limitations of the study are worth mentioning, first, although the focus of this study was to evaluate the etiology of meningitis, onset of symptoms and accompanying treatment strategies were not captured. This was a small study which may not be representative nationally for all of Kenya.

## Conclusion

Bacteria causing meningitis was isolated especially among younger and some unvaccinated (1 to 4 years) patients. This calls for the improvement and adoption of modern methods to diagnosis the etiological agents of meningitis in order to offer proper management meningitis especially in children given the documented difficulties in distinguishing bacterial meningitis clinically in this age group because of the no-specificity features of bacterial meningitis. Although our study did not serotype bacterial isolates, a number of cases were unvaccinated, therefore immunization against bacterial pathogens should be ensured.

### What is known about this topic

- *S. pneumonia*, *N. meningitides* and *H. influenza*e are the most common bacterial pathogens associated with bacterial meningitis;
- Use of meningitis vaccine is a good prevention strategy of bacterial meningitis but it is not included in routine immunization schedule in the country.

### What this study adds

- It is discovered in this study that *S. pneumonia* is the most common cause of bacterial meningitis in this sub-urban population.

## Competing interests

The authors declare no competing interest.

## References

[1] Brouwer MC, Tunkel AR, van de Beek D (2010). Epidemiology, diagnosis, and antimicrobial treatment of acute bacterial meningitis. Clin Microbiol Rev.

[2] Lukšić I (2013). Estimating global and regional morbidity from acute bacterial meningitis in children: assessment of the evidence. Croat Med J.

[3] Greenhow TL, Hung YY, Herz AM, Losada E, Pantell RH (2014). The changing epidemiology of serious bacterial infections in young infants. Pediatr Infect Dis J.

[4] Ghotaslou R (2012). Detection of acute childhood meningitis by PCR, culture and agglutination tests in Tabriz, Iran. Acta Medica Iranica.

[5] Mahmoudi S (2013). Acute bacterial meningitis among children admitted into an Iranian referral children's hospital. Japanese Journal of Infectious Diseases.

[6] Mishal J (2008). Community acquired acute bacterial meningitis in children and adults: an 11-year survey in a community hospital in Israel. European Journal of Internal Medicine.

[7] Rossi PG (2009). Incidence of bacterial meningitis (2001-2005) in Lazio, Italy: the results of a integrated surveillance system. BMC Infectious Diseases.

[8] Cohn AC (2010). Changes in Neisseria meningitidis disease epidemiology in the United States, 1998-2007: implications for prevention of meningococcal disease. Clin Infect Dis.

[9] Muller LM, Gorter KJ, Hak E, Goudzwaard WL, Schellevis FG, Hoepelman AI, Rutten GE (2005). Increased risk of common infections in patients with type 1 and type 2 diabetes mellitus. Clin Infect Dis.

[10] Scott JA (2007). The preventable burden of pneumococcal disease in the developing world. Vaccine.

[11] Conklin LM (2016). High Streptococcus pneumoniae colonization prevalence among HIV-infected Kenyan parents in the year before pneumococcal conjugate vaccine introduction. BMC Infect Dis.

[12] Veltman JA, Bristow CC, Klausner JD (2014). Meningitis in HIV-positive patients in sub-Saharan Africa: a review. J Int AIDS Soc.

[13] Berkley JA (2001). Diagnosis of acute bacterial meningitis in children at a district hospital in sub-Saharan Africa. Lancet.

[14] Berkley JA (2005). Use of clinical syndromes to target antibiotic prescribing in seriously ill children in malaria endemic area: observational study. BMJ.

[15] Garcia LS (2010). Clinical microbiology procedures handbook.

[16] Klugman KP, Madhi SA, Feldman C (2007). HIV and pneumococcal disease. Current opinion in infectious diseases.

[17] Mwaniki MK (2011). Clinical indicators of bacterial meningitis among neonates and young infants in rural Kenya. BMC Infectious Diseases.

[18] Karanja BW (2013). Risk factors for hearing loss in children following bacterial meningitis in a tertiary referral hospital. International Journal of Otolaryngology.

[19] Abro AH (2016). Sphingobacterium multivorum Bacteremia and Acute Meningitis in an immunocompetent adult patient: a case report. Iranian Red Crescent Medical Journal.

[20] Sandhu GK (2011). Tuberculosis: Current Situation, Challenges and Overview of its Control Programs in India. J Glob Infect Dis.

[21] Kali A (2016). Aeromonas hydrophila meningitis and fulminant sepsis in preterm newborn: a case report and review of literature. Indian Journal of Medical Microbiology.

[22] Farrugia MK (2016). Candida meningitis in an immunocompetent patient detected through (1-3)-beta-d-glucan. International Journal of Infectious Diseases.

[23] Cabrerizo M (2008). Molecular epidemiological study of HEV-B enteroviruses involved in the increase in meningitis cases occurred in Spain during 2006. Journal of medical virology.

[24] Ryu J-U (2014). Outbreaks of mumps: an observational study over two decades in a single hospital in Korea. Korean J Pediatr.

[25] Talbert AW (2010). Invasive bacterial infections in neonates and young infants born outside hospital admitted to a rural hospital in Kenya. Pediatric Infectious Disease Journal.

[26] Molesworth AM (2002). Where is the meningitis belt? Defining an area at risk of epidemic meningitis in Africa. Trans R Soc Trop Med Hyg.

[27] Mueller JE, Gessner BD (2010). A hypothetical explanatory model for meningococcal meningitis in the African meningitis belt. International Journal of Infectious Diseases.

[28] Feikin DR (2010). High rate of pneumococcal bacteremia in a prospective cohort of older children and adults in an area of high HIV prevalence in rural western Kenya. BMC Infectious Diseases.

[29] MenAfriCar consortium (2015). The Diversity of Meningococcal Carriage Across the African Meningitis Belt and the Impact of Vaccination With a Group A Meningococcal Conjugate Vaccine. J Infect Dis.

[30] Özlem NE, Akinci A, Bilir P (2011). Tuberculous Meningitis Associated with Diabetic Ketoacidosis. J Clin Res Pediatr Endocrinol.

[31] Aydin Y (2005). Herpes simplex type-2 encephalitis masked by diabetic ketoacidosis. J Natl Med Assoc.

